# Benchmarking of numerical integration methods for ODE models of biological systems

**DOI:** 10.1038/s41598-021-82196-2

**Published:** 2021-01-29

**Authors:** Philipp Städter, Yannik Schälte, Leonard Schmiester, Jan Hasenauer, Paul L. Stapor

**Affiliations:** 1 Institute of Computational Biology, Helmholtz Zentrum München - German Research Center for Environmental Health, 85764 Neuherberg, Germany; 2 Center for Mathematics, Technische Universität München, 85748 Garching, Germany; 3 Faculty of Mathematics and Natural Sciences, University of Bonn, 53113 Bonn, Germany

**Keywords:** Computer modelling, Computer science, Differential equations, Dynamical systems, Nonlinear dynamics, Numerical simulations, Software, Biochemical reaction networks, Computational models, Computational platforms and environments, Software

## Abstract

Ordinary differential equation (ODE) models are a key tool to understand complex mechanisms in systems biology. These models are studied using various approaches, including stability and bifurcation analysis, but most frequently by numerical simulations. The number of required simulations is often large, e.g., when unknown parameters need to be inferred. This renders efficient and reliable numerical integration methods essential. However, these methods depend on various hyperparameters, which strongly impact the ODE solution. Despite this, and although hundreds of published ODE models are freely available in public databases, a thorough study that quantifies the impact of hyperparameters on the ODE solver in terms of accuracy and computation time is still missing. In this manuscript, we investigate which choices of algorithms and hyperparameters are generally favorable when dealing with ODE models arising from biological processes. To ensure a representative evaluation, we considered 142 published models. Our study provides evidence that most ODEs in computational biology are stiff, and we give guidelines for the choice of algorithms and hyperparameters. We anticipate that our results will help researchers in systems biology to choose appropriate numerical methods when dealing with ODE models.

## Introduction

Systems biology aims at understanding and predicting the behavior of complex biological processes through mathematical models^1^. In particular, ordinary differential equations (ODEs) are widely used to gain a holistic understanding of the behaviour of such systems^2^. These ODE models are often derived from biochemical reaction networks and stored/exchanged using the Systems Biology Markup Language (SBML)^3^ or Cell Markup Language (CellML)^4^. Based on these community standards, large collections of published ODE models have been made available to enhance reproducibility of scientific results, like BioModels^5^ and JWS^6^. These model collections allow an analysis of typical properties of published ODE models of biological processes and are an excellent source for method studies and method development^7^.

Most published models of biochemical reaction networks are non-linear and closed form solutions are not available. Accordingly, numerical integration methods have to be employed to study them^8^. For this task, the modeler has the choice between many possible numerical simulation algorithms, which have specific hyperparameters that can strongly impact the result. Key parameters are for instance the relative and absolute tolerance, which determine the precision of the numerical solution. Too relaxed tolerances may lead to incorrect results, while too strict tolerances may result in an unnecessarily high computation time, or may even lead to a failure of ODE integration, if the desired solution accuracy cannot be achieved^9^.

Various theoretical results about the reliability and the scaling behaviour of ODE solvers are available^10^. However, to the best of our knowledge, there is no comprehensive study on the impact of ODE solver settings on the simulation results and their reliability which focuses on models of biological processes. So far, case studies using only single models or a very small number of models have been carried out, which demonstrate the need for efficient implementations of ODE solvers (see, e.g.,^8^). In addition to this, various hypotheses on the general properties of ODE models in systems biology exist, e.g., whether or not the underlying ODEs are expected to be stiff—an ODE is (informally) called stiff, if it exhibits different time-scales, i.e., fast and slow dynamics are described at the same time^11,12^. The absence of representative studies and statistical evaluations is surprising as various tasks require large numbers of numerical simulations, rendering computation efficiency and numerical robustness an important topic. In particular when performing parameter estimation, a model has to be simulated, i.e., the underlying ODE has to be solved, thousands to millions of times^8,13^. Indeed, it has recently been pointed out that the ODE solver is actually a crucial hyperparameter, which often remains unconsidered^14^. Hence, studying these questions on a wide set of real world applications is of high importance for many modeling applications.

In this work, we benchmark numerical integration methods and their hyperparameters. We established a benchmark collection of 142 models from the two freely accessible databases BioModels^5,15^ and JWS Online^6^, which covers a broad range of different properties. These models were simulated using various ODE solver algorithms implemented in the ODE solver toolboxes CVODES^16^ from the SUNDIALS suite^9^ and the ODEPACK package^17^, which are possibly the most widely used software package to integrate ODEs in systems biology. We investigated various combinations of ODE integration algorithm, non-linear solver employed in implicit multi-step methods, linear solvers employed within the non-linear solver, and relative and absolute error tolerances. By analyzing the computation time and the failure rate, we derived guidelines for the tuning of ODE solvers in systems biology, which facilitate fast and reliable simulation of the corresponding ODE systems.

## Results

To analyze combinations of algorithms and hyperparameters, we considered the ODE solvers from the SUNDIALS package CVODES^9,16^, which implement implicit multi-step methods for numerically solving an initial value problem, i.e., an ODE with initial conditions and offer a variety of hyperparameters. We furthermore included the ODEPACK^17^ package in our analysis, which uses the multi-step algorithm LSODA, which adaptively switches between methods stiff and nonstiff ODEs for numerical integration^18^. CVODES and ODEPACK are used in multiple systems biology toolboxes^19–22^ and are therefore particularly relevant.

An initial value problem is solved by iterative time-stepping, following a specific integration algorithm^10,23^, (see Methods, Numerical integration methods for ODEs for more details). In each time step, a non-linear problem is solved via a fixed-point iteration or a sequence of linear problems. These are solved until a previously defined precision, given by absolute and relative error tolerances, is fulfilled.

CVODES offers the following hyperparameters:

1. Integration algorithm:
  - Adams-Moulton (AM): implicit multi-step method of variable order 1 to 12, chosen automatically
  - Backward Differentiation Formula (BDF): implicit multi-step method of variable order 1 to 5, chosen automatically
2. Non-linear solvers:
  - Functional: solution to the non-linear problem directly via a fixed-point method
  - Newton-type: linearization of the non-linear problem
3. Linear solver (only when using Newton-type non-linear solver):
  - DENSE: dense LU decomposition
  - GMRES: iterative generalized minimal residual method on Krylov subspaces
  - BICGSTAB: iterative biconjugate gradient method on Krylov subspaces
  - TFQMR: iterative quasi-minimal residual method on Krylov subspaces
  - KLU: sparse LU decomposition
4. Error tolerances: upper bounds for the absolute and relative error made in each time-step

ODEPACK offers absolute and relative error tolerances as only hyperparameters, with LSODA switching automatically between a BDF algorithm using a Newton-type method with dense LU decomposition in the stiff and an AM algorithm using a functional iterator in the nonstiff case.

We will call a combination of these hyperparameters a solver setting.

As it is still unclear which solver settings are best suited for models of biochemical reaction networks, we performed a comprehensive empirical study to answer this question. Therefore, we considered for CVODES all 20 possible combinations of integration algorithm, non-linear solver, and linear solver with 7 different error tolerances combinations. For ODEPACK, we used the same 7 combinations of error tolerances. Furthermore, we considered 36 error tolerance combinations in an in-depth tolerance study for CVODES, yielding in total 176 ($=20 \times 7 + 7 + (36 - 7)$) different solver settings.

As performance characteristics of a solver setting, we consider the following two criteria:

1. Integration failures: These failures may occur if either the dynamics of the system become too stiff or if the state of the system diverges. In these cases, the requested numerical accuracy per integration step cannot be achieved by a solver setting and the (adaptively chosen) step-size falls below machine precision and integration gets stuck.
2. Computation times: The total computation time is determined by the number of steps the solver takes and their individual computation times. Both quantities can vary heavily depending on the solver settings.

### A comprehensive model collection allows the systematic benchmarking of hyperparameters

**Figure 1:**
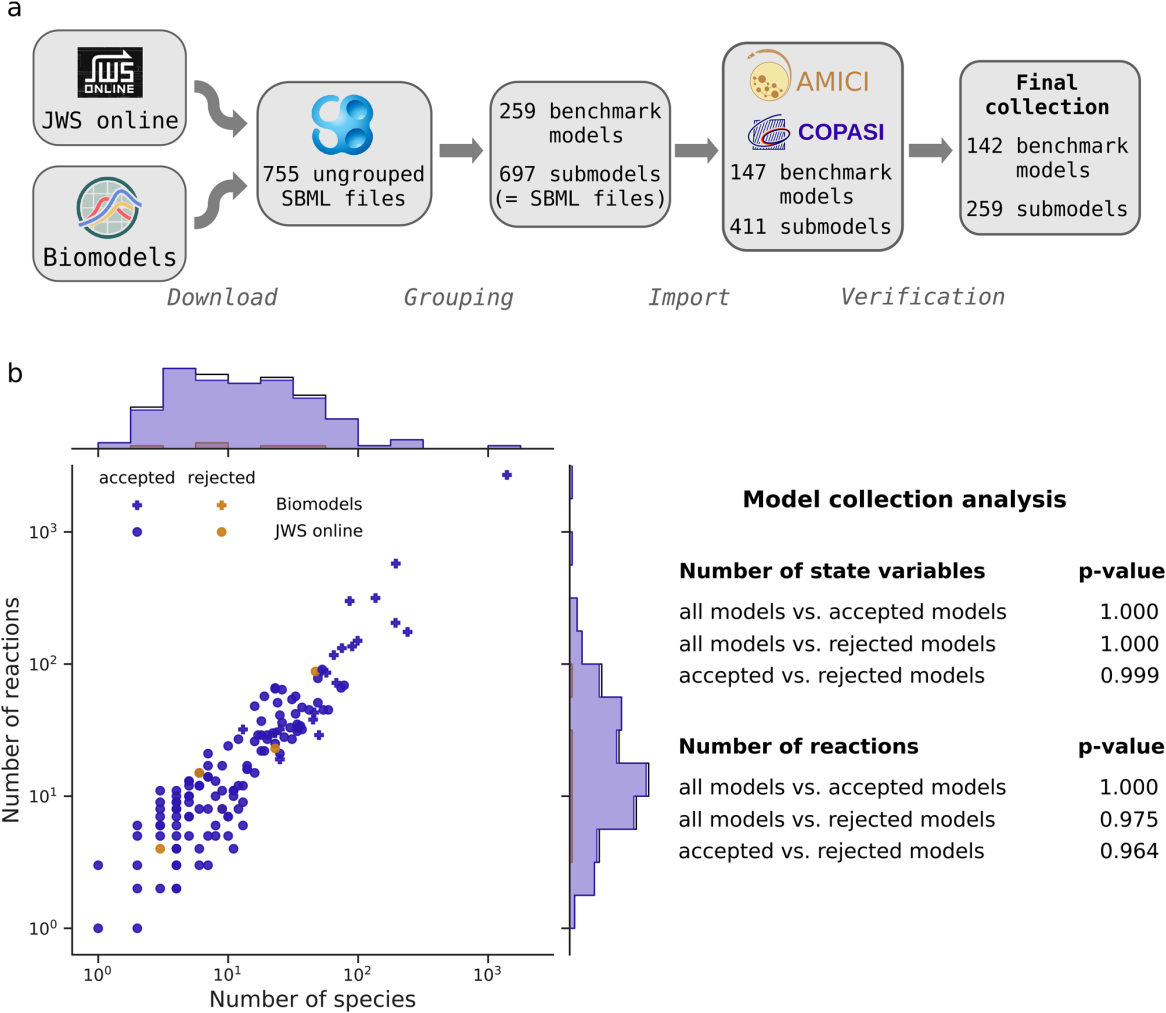
Model collection. (**a**) Workflow of collecting models for the benchmark collection. Models were downloaded, grouped according to their number of species and reactions, the author name and year of their publication, imported with AMICI and COPASI, and simulation results were compared to reference trajectories using multiple solver settings. (**b**) Basic properties (number of state variables and reactions) of the benchmark collection models, as joint scatter plot, indicating the number of accepted, rejected, and the total number of grouped models. The distributions of the numbers of state variables and numbers of reactions were compared for all imported models, vs. accepted models vs. rejected models by a Kolmogorov-Smirnov test, to assess a possible bias due to the filtering step.

For a comprehensive study on a variety of ODE models, we downloaded all SBML models from the JWS database^6^. As all of those models had less than 100 state variables, we complemented them with a set of the largest models from the BioModels database^5^. To simulate these models using CVODES, we imported them with AMICI (Advanced Multi-language Interface to CVODES and IDAS^22^), which performs symbolic preprocessing and creates and compiles executable code for each model. For simulation with the LSODA algorithm from ODEPACK, we furthermore imported these model with COPASI^19^. As neither AMICI nor COPASI support all features of SBML, only a subset of the models could be imported with both toolboxes. These SBML models were then grouped together, to avoid counting variations of the same model multiple times, yielding 148 benchmark models comprising 411 SBML files: One benchmark model may hence consist of multiple submodels, i.e., SBML files. Computation times were averaged over the submodels of a benchmark model. We considered the simulation of a benchmark model failed if the simulation of at least one of its submodels failed. To ensure a proper comparison, we verified the correctness of the simulated trajectories (Fig. 1a, Supplementary Fig. S1), based on reference solutions. After this filtering step, we were left with 142 benchmark models comprising 259 submodels, of which a majority comprises between 10 and 100 state variables and reactions (Fig. 1b). A Kolmogorov-Smirnov test on the distributions of the numbers of model species and reactions comparing the accepted with the rejected models yielded little evidence for these distributions to be different (p-values of 0.999 and 0.964 for species and reactions, respectively). For details on the construction of the benchmarking collection (e.g., source of reference solutions) we refer to the Methods, Creation of the ODE solver benchmark collection.

### Newton-type method outperforms functional iterator when solving the non-linear problem

Using the 142 benchmark models, we analyzed the impact of different hyperparameters on the performance of numerical ODE solvers. As we expected the combination of the non-linear and linear solver to strongly impact the reliability of the numerical ODE integration, we decided to first study this aspect.

We compared the rate of integration failure for the two available non-linear solvers—the functional iterator and the Newton-type solver—for both ODE integration algorithms in CVODES (AM and BDF), all available linear solvers and a set of heterogeneous error tolerances, motivated by commonly used ODE solver settings. LSODA uses a fixed implementation for solving the nonlinear problem which can not be altered.

**Figure 2:**
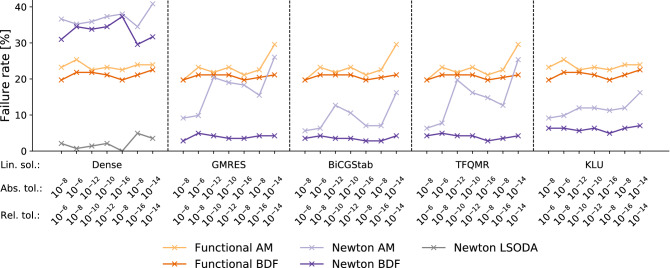
Non-linear solver. Comparison of functional and Newton-type non-linear solver in terms of failure rate. Each point represents the failure rate when simulating all models for a given combination of integration algorithm, non-linear solver, linear solver and error tolerances.

This comparison revealed that the Newton-type method performs better than the functional iterator: While the functional iterator, which does not rely on a linear solver, failed on average for 10–15% of the models, the Newton-type method failed substantially less (Fig. 2). Indeed, in combination with the BDF integration algorithm, we observed for all but the dense direct linear solver DENSE a failure rate of roughly $5\%$ of the models. Overall, the BDF integration algorithm appeared to be less prone to integration failure than the AM method. Fisher’s exact tests showed that the difference was not significant for the functional iterator (with a p-value of 0.215 for the linear solver KLU), but significant when using the Newton-type method (p-value of $1.35 \cdot 10^{-5}$ for KLU). The LSODA algorithm outperformed the best results of the BDF algorithm and the Newton-type method (p-values using Fisher’s exact test of $7.43\cdot 10^{-6}$ for LSODA vs. KLU and 0.025 for LSODA vs. BICGSTAB). This is surprising, as it uses a Newton-type method with dense linear solver and relies on the BDF algorithm—a setting which performed clearly worse in CVODES. This suggests that the implementation of the non-linear and linear solver in ODEPACK is superior to the one in CVODES for the considered models class.

These results can be considered as hint that many models are stiff, as the AM algorithm is less tailored to models exhibiting stiff dynamics^23^ than the BDF or the LSODA algorithm.

Interestingly, some models could only be integrated using an iterative linear solver, while another small set of models could only be integrated using a direct linear solver. This suggests that it may be crucial to have the choice between different linear solvers in certain applications. Furthermore, we observed that the failure rate tended to increase for stricter error tolerances. The highest failure rate was observed for the Newton-type method in combination with the dense linear solver. For all other linear solvers, the Newton-type method is less prone to integration failure than the functional iterator and also more efficient in terms of computation time (Supplementary Fig. S2). Thus, we focus in the following only on results for the Newton-type method.

### The sparse direct linear solver from CVODES scales best

**Figure 3:**
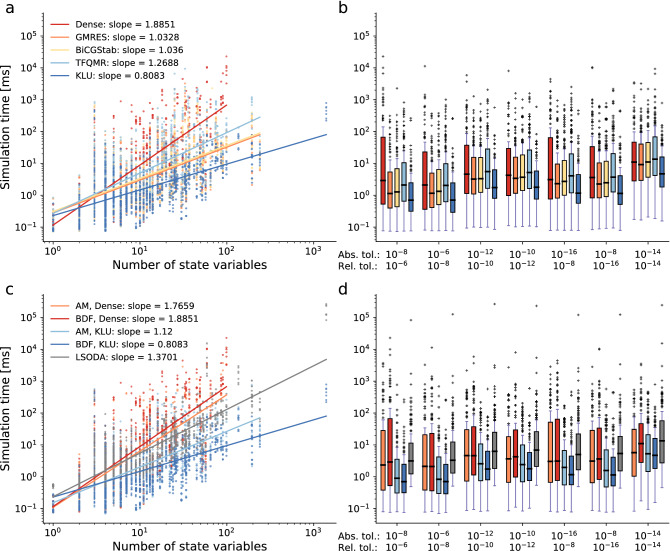
Linear solvers. Scaling behavior and computation time comparison of the linear solvers and integration algorithms. (**a**) Each point depicts the simulation time of one model (median of 25 repetitions) with one solver setting: BDF integration algorithm, Newton-type non-linear solver, one tolerance combination, and one linear solver (which are color-coded). Results are shown for seven tolerance combinations and five linear solvers, meaning there are 35 points for each model. The accompanying linear regressions display the scaling behavior with respect to the number of state variables. (**b**) Box plot of the simulation times, separated by the tolerance combination in addition to the linear solver, using the BDF integration algorithm and the Newton-type non-linear solver. (**c**) Scaling behavior of the different integration algorithms using the Newton-type method and the direct linear solvers with the same setup as in subfigure (**a**). (**d**) Box plot of the simulation times for the different integration algorithms using the Newton-type method and the direct linear solvers with the same setup as in subfigure b.

To determine the performance of the different linear solvers, we assessed their computation times. For the BDF algorithm, we found that the dense direct solver exhibited the worst scaling behavior with respect to the number of state variables, followed by the iterative solver TFQMR (Fig. 3a). The iterative linear solvers GMRES and BICGSTAB showed a roughly linear scaling behavior for the BDF algorithm of the computation time with the model size. The sparse direct solver KLU had the best scaling behavior. For the BDF algorithm with the linear solver KLU, the complexity of the numerical integration increased roughly by a factor of four when the model size increased by a factor of five.

Assessing the computation times across models for different error tolerances confirmed that the sparse direct linear solver KLU performed best for every tolerance combination (Fig. 3b, Supplementary Figs. S3, S4, and S5), followed by the two iterative linear solvers GMRES and BICGSTAB, which showed comparable computation times. The dense linear solver DENSE yielded the highest computation times. As expected, stricter error tolerances consistently led to an increase of computation time for all employed solver settings.

When comparing the scaling behavior of the direct linear solvers across integration algorithms, we saw that KLU performed best and DENSE performed worst (both from CVODES, Fig. 3c), while the LSODA implementation had an intermediate scaling. Overall, the combination of BDF and KLU was the best performer, also in terms of computation time (Fig. 3d). In general, the scaling behavior for the AM integration algorithm was slightly worse than for the BDF algorithm (Supplementary Figs. S3, S4, and S5).

### Choosing error tolerances is a trade-off between accuracy, reliability, and computation time

In a next step, we studied the effect of the error tolerances on the computation time. As the Newton-type non-linear solver with the sparse direct linear solver KLU outperformed the other combinations, we used this combination to analyze the impact of the absolute and relative error tolerances on computation time and failure rate. For an extensive tolerance study, not only the hitherto seven, but 36 tolerance combinations were analyzed, covering a broad spectrum. As upper bounds for the relative and absolute tolerances we used $10^{-6}$, as more relaxed tolerances provided an insufficient agreement with the reference trajectories (see Methods, Creation of the ODE solver benchmark collection and Supplementary Fig. S1).

**Figure 4:**
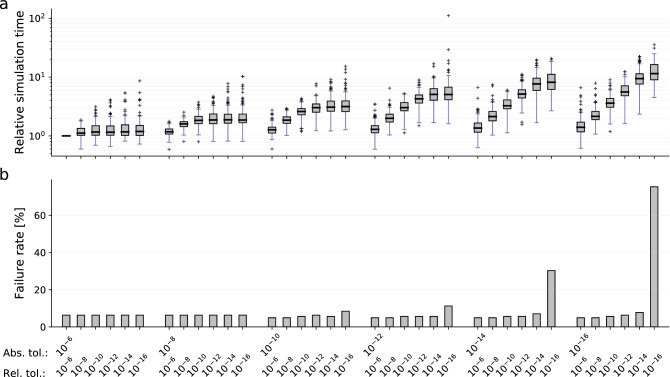
Integration error tolerances. Comparison of 36 combinations of absolute and relative error tolerances. All models were simulated using the BDF integration algorithm, the Newton-type non-linear solver, and the linear solver KLU, for each tolerance combination. (**a**) Relative simulation times, normalised by those for absolute and relative error tolerances $(10^{-6}, 10^{-6})$. (**b**) The corresponding failure rates.

To compare computation times across all models, we analyzed CPU time ratios. The computation time for each model and each error tolerance combination was normalized by the computation time for the most relaxed tolerance combination, i.e., absolute and relative tolerance of $10^{-6}$ (Fig. 4a, Supplementary Fig. S6). We found that for most models, the computation time increases with the enforced accuracy. Yet, we found some models, which were simulated faster when mildly restricting the error tolerances, indicating a non-trivial relation. Apart from those exceptions, the median CPU time increased monotonically by roughly an 11-fold when restricting the absolute and relative error tolerances from $10^{-6}$ to $10^{-16}$. Hence, restricting the requested error tolerances by ten orders of magnitude increased the median computation time by about one order of magnitude, which was less than we had expected. Furthermore, strict relative error tolerances tended to have a bigger impact on the computation time and the failure rate than strict absolute tolerances. While the overall failure rate mildly decreased when restricting the error tolerances to a range between $10^{-8}$ and $10^{-12}$, it increased markedly when both error tolerances were set to very strict values at the same time (Fig. 4b).

### Fully implicit algorithms are the best choice for most models

Since we have seen noticeable differences in their behavior, we compared the integration algorithms AM, BDF, and LSODA for each model individually regarding their computation time and failure rate. Therefore, we used again the seven tolerance combinations which we employed for the analysis of the non-linear and the linear integration algorithms, together with the Newton-type non-linear solver and the KLU linear solver for AM and BDF.

**Figure 5:**
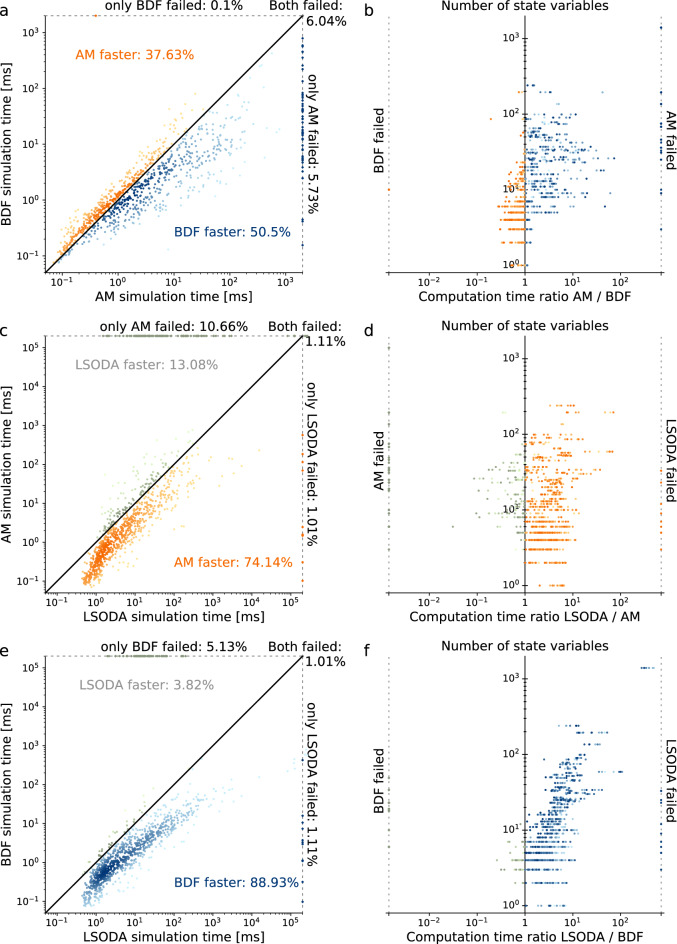
Comparison of integration algorithm (**a**) Each scatter point shows the computation time for a model using the AM (x-axis) or BDF (y-axis) algorithm with the Newton-type non-linear solver, the linear solver KLU and one out of the seven tolerance combinations. Darker colors represent a higher scatter point density. (**b**) Computation time for AM divided by the computation time for BDF with respect to the number of state variables, using the color coding from subfigure A. (**c**) Comparison of the LSODA algorithm (x-axis) with the AM algorithm (y-axis) with the same setup as in a. (**d**) Computation time ratios for the LSODA devided by the AM algorithm, using the color coding from c. (**e**) Comparison of the LSODA algorithm (x-axis) with the BDF algorithm (y-axis) with the same setup as in a. (**f**) Computation time ratios for the LSODA devided by the BDF algorithm, using the color coding from e.

We observed that for most models and settings, the BDF algorithm was faster than the AM algorithm (roughly $50 \%$ vs. $37 \%$, Fig. 5a). For a number of models, especially for the larger ones, BDF was faster by almost two orders of magnitude when compared to AM. In contrast, AM outperformed BDF by at most a factor of 5 (Fig. 5b). Importantly, the BDF algorithm was not only computationally more efficient, but showed also the lower failure rate: For about $6 \%$ of the settings, both algorithms failed to integrate the ODE. For an additional $5.7 \%$ of the cases, BDF could still integrate the ODE although AM failed, while the opposite was true in only $0.1 \%$ of the cases. Hence, the AM algorithm failed about twice as often to integrate the ODE system as the BDF algorithm.

When comparing AM and BDF with LSODA, we found that for the majority of models, AM and BDF markedly outperformed LSODA in terms of computation time: AM was faster for $74 \%$ of the models (vs. $13 \%$ for LSODA, (Fig. 5c,d) and BDF was faster for $89 \%$ of the models (vs. $3.8 \%$ for LSODA, (Fig. 5e,f). For certain models, AM outperformed LSODA by up to a 70-fold, while LSODA outperformed AM by up to a 30-fold. BDF was faster by up to a 500-fold, while LSODA achieved to outperform BDF by at most a 4-fold. In particular for models with more than 15 state variables, BDF was always faster than LSODA. A possible explanation for the lower computation times of the algorithms implemented in CVODES is the sparse linear solver in the Newton-type method, and the fact that AMICI provides an analytically calculated Jacobian matrix to CVODES, whereas ODEPACK computes the Jacobian by finite differences in COPASI, which is computationally more demanding for larger systems.

It is important to note that AM and BDF suffered from higher rates of integration failure than LSODA: BDF failed almost three times as often as LSODA ($6.1 \%$ vs. $2.1 \%$), AM about more than five times as often ($11.8 \%$ vs. $2.1 \%$). However, these failure rates improved for AM and BDF when using one of the iterative linear solver BICGSTAB (AM: $9.3 \%$, BDF: $3.5 \%$), such that BDF was almost on par with LSODA (Supplementary Fig. S7). Using the linear solver GMRES, BDF still showed a good performance, while AM worked less well (Supplementary Fig. S8). Hence, LSODA is overall clearly slower than the CVODES implementation of BDF with its sparse linear solver, but more robust to integration failure.

## Discussion

Modeling with ODEs is among the most popular approaches to develop a holistic understanding of cellular processes in systems biology. Here, we collected a set of 142 benchmark ODE models collected from publications and used them to carry out a comprehensive study on the most essential hyperparameters of numerical ODE solvers. To the best of our knowledge, this is the first extensive study focusing on ODE integration itself, investigating a total of 176 ODE solver settings. The use of a large number of established models makes it highly relevant to the community. Although the optimal choice of hyperparameters is model dependent, our findings allow to draw some general conclusions:

Firstly, we found that, in general, error tolerances should not be relaxed beyond the value of $10^{-6}$, as otherwise simulation results tend to deviate markedly from results of more accurate computations. However, too strict error tolerances substantially increase the computation time and, more importantly, can lead to failure of ODE integration. We conclude that for most models, error tolerances between $10^{-8}$ and $10^{-14}$ are a reasonable choice, with absolute error tolerances being stricter than relative error tolerances, at least for the ODE solver implementation considered in this study.

Secondly, we observed that for more than $60\%$ of the models, the BDF integration algorithm was superior to the AM integration algorithm in the implementation of CVODES. As the AM algorithm is generally recommended for mildly stiff problems and BDF is more tailored to stiff systems^23^, this implies that most ODE models in systems biology show substantial stiffness. Stiffness was already hypothesized for ODEs arising from biological systems^11,12^, but to the best of our knowledge, this was never quantified. Hence, our results suggest that fully implicit methods for ODE integration with adaptive time-stepping and error control are necessary to obtain reliable results, except for special cases, where clear motivations for other approaches can be given. We also want to stress that all ODE models were analyzed at the reported parameter values, for which ODE integration is supposed to work well. However, these parameters often have to be estimated first, by evaluating the ODE at various parameters and comparing model simulations with measurement data^13,24^. During this estimation process, the model does often not yet reflect a realistic behavior and in our experience stiffness is encountered substantially more often. Hence, in parameter estimation, stiffness is likely to be even more present.

Thirdly, when comparing algorithms for solving the non-linear and the linear problem within implicit integration methods, we found that the fastest setting is using a Newton-type method for the non-linear problem and a sparse direct solver for the linear problem (in our case KLU), in particular with a better scaling behavior towards higher-dimensional models. This setting was also among the most reliable settings when comparing ODE integration failure, but was outperformed by iterative linear solvers and by the LSODA implementation from ODEPACK. In contrast, the dense direct linear solver from the CVODES implementation showed the worst performance. Furthermore, it was pointed out that for stiff ODE systems, Newton-type non-linear solvers tend to be superior to fixed-point methods^25^, which we can underline by our findings.

In our opinion, a good default setting for most models should be an ODE solver using the BDF (or LSODA) algorithm, together with a Newton-type approach for the non-linear, and a sparse direct solver for the linear problem. If this leads to integration failure, an iterative linear solver—in our case, BICGSTAB worked best—may be a promising alternative. It may be helpful to check for a given model whether the AM integration algorithm reduces the computation time, as this is generally model dependent. However, model simulations should then ideally be verified using a BDF algorithm to ensure accuracy.

While this study focused on numerically solving ODE systems, a valuable extension would be assessing the performance of ODE integration when performing it with forward and adjoint sensitivity analysis^26^. This is a typical setting when estimating unknown model parameters, as sensitivities are needed to compute the gradient of an objective function which depends on the ODE solution^13^. It would be particularly interesting to see whether it could be inferred when forward and when adjoint sensitivity analysis should be used: Although forward sensitivity analysis is more commonly used and may be superior for small models, adjoint sensitivity analysis is known to be more efficient for large models^26,27^. Yet, no clear rule for deciding between those two approaches could be derived so far. The respective sensitivity equations may also change in particular the stiffness of the ODE, which may impact optimal hyperparameters.

Moreover, the multi-step algorithms in CVODES and ODEPACK could be compared to other approaches, such as the (implicit) single-step algorithms which are provided in, e.g., the toolbox FATODE^28^. Another direction of future research may be accelerating computations via GPUs, which can be advantageous for certain use cases^29^. Additionally, it should be noted that many ODE solver toolboxes allow to specify error tolerances for each state variable of the ODE system separately. If prior knowledge on the trajectories of the model simulation is available, this option may be exploited to either improve accuracy or speed up model simulation.

In conclusion, we are certain that the presented study will be helpful for both modelers and toolbox developers, when choosing hyperparameters for ODE solvers to yield both reliable and efficient simulations.

## Methods

### Numerical integration methods for ODEs

An ODE model describes the dynamics of a vector of state variables $x(t) \in \mathbb {R}^{n_x}$ with respect to time *t*, model parameters $\theta$, and input parameters *u*. The time evolution is given by a vector field *f*:

1 $$\begin{aligned} \frac{d}{dt} x(t) = f(x(t), \theta , u) , \quad x(0) = x_0(\theta , u). \end{aligned}$$

As typically the solution *x*(*t*) cannot be computed analytically, we consider in this study different numerical integration methods which proceed along the time course step by step: Starting from time $t_0$ and state $x_0$, an approximation $x_k$ of the state $x(t_{k})$ at the *k*-th time-step $t_k$ is computed from the states and the vector fields at previous time points. In so-called multi-step methods, the *s* previous time-steps of the ODE solver $t_{k-1}, \ldots , t_{k-s}$ are used, where $s\in \mathbb {N}$ fixes the order of the method. The most general form of a multi-step method is:

2 $$\begin{aligned} x_k = \sum \limits _{j=1}^s \alpha _j x_{k-j} + h_{k} \sum \limits _{j=0}^s \beta _j f(t_{k-j}, x_{k-j}, \theta , u) \end{aligned}$$

The difference $h_{k} = t_k - t_{k-1}$ defines the time-step length, and the coefficients $\alpha _j$ and $\beta _j$ determine the exact algorithm. When $\beta _{0} = 0$, the method is explicit, otherwise implicit. Generally, implicit algorithms are considered to be better suited for ODE systems with so-called stiff dynamics^23^.

For implicit methods and non-linear vector fields *f*, a non-linear problem has to be solved to determine $x_{k}$. In order to solve the non-linear problem, either a fixed-point iteration can be used, which tries to approximate $f(t_{k}, x(t_{k}), \theta , u)$ step by step, or a Newton-type method can be employed, which reduces Equation (2) to a series of linear problems, which can be denoted as:

3 $$\begin{aligned} \left( \mathbb {I} - \gamma \nabla _x f(t_k, x_k, \theta , u) \right) \Delta x_k = - f(t_k, x_k, \theta , u), \end{aligned}$$

where is the unit matrix $\mathbb {I}$, $\gamma > 0$ a relaxation constant and $\nabla _x f(t_k, x_k, \theta , u)$ the Jacobian of the right hand side. In the latter case, algorithms for solving linear problems have to be used repeatedly. Here, we consider two approaches: Direct approaches, which try to solve Equation (3) by factorization of the matrix $\mathbb {I} - \gamma \nabla _x f(t_k, x_k, \theta , u)$ (such as LU factorization); and iterative approaches, such as Krylov subspace methods, which try to compute *x* by a sequence of improving approximations $x^0, x^1, \ldots , x^m$, until $x^m$ solves Equation (3) up to a previously defined accuracy.

### Statistical tests

To perform the statistical tests, we used the implementation of the stats package in the SciPy toolbox (release 1.4.1). The Kolmogorov-Smirnov tests were performed as a two-sample test using the function ks_2samp, Fisher’s exact tests were performed using the function fisher_exact.

### High performing software packages for ODE integration

It has been shown in several studies that it is critical to use high-performance ODE solver toolboxes written in compiled languages such as C++ or FORTRAN, since those have shown to reduce computation time by two to three orders of magnitude^8,30^. For this reason, we focused on the C-based ODE solvers in CVODES, which is part of the SUNDIALS solver package^9^ and the LSODA algorithm from the FORTRAN-based ODEPACK suite. These solvers are used in several established toolboxes, e.g., Data2Dynamics^20^, libroadrunner^21^, COPASI^19^, or AMICI^22^ and represent probably the two most widely used implementations in the field of systems biology. Appropriate alternatives might be in our opinion other solver toolboxes written in compiled languages, such as the FORTRAN-based FATODE implementation^28^ or the FORTRAN-based ODEPACK implementation^17^, or reimplementations of these. Additionally, Julia written toolboxes, such as DifferentialEquations.jl could also be valuable alternatives^31^. For applications which benefit from a high degree of parallelization, GPU acceleration may also be beneficial. Approaches in this direction are implemented in, e.g., the toolboxes LASSIE^32^ and cupSODA^29,33^.

### Setup of the numerical experiments

All numerical simulations were repeated 25 times to avoid outliers in the computation time. Afterwards, the median values of all these computation times were stored. Since a repeated simulation using the same solver setting does not change the success of the simulation, this information was stored after the first simulation. In total, 49,984 data points for computation time and success of the simulation were created. These were then analyzed and displayed in different ways to illustrate the impact of the hyperparameters on simulation time and reliability. The whole dataset and the code of the implementation is made available at 10.5281/zenodo.4013853 and as a repository on GitHub at https://github.com/ICB-DCM/solverstudy. The study was performed on a laptop with Ubuntu 20.04.1 LTS operating system, an Intel Core i7-8565U CPU with a maximal capacity of 4.60GHz, and 40GB RAM.

### Creation of the ODE solver benchmark collection

As a first step, we downloaded all models from the JWS database (date of download: June 17, 2019). As these models were comparably small, we complemented this collection by a set of larger models from the BioModels database (date of download: June 24, 2019) and a particularly large SBML model of cancer signalling^34^, available at https://github.com/ICB-DCM/CS_Signalling_ERBB_RAS_AKT (commit 365e0be, date of download: June 24, 2019). These larger models allowed us to also assess the scaling behavior of computation times with respect to the size of the ODE models. In total, we thus downloaded 755 SBML models.

On JWS, the models are grouped as SED-ML models^35^. A SED-ML model consists of possibly multiple SBML (sub-)models, may contain changes to be performed on the SBML (sub-)models, and contains time frames for specific simulations. The SBML models coming from JWS were hence adapted according to the specifications in the SED-ML files and simulation time frames were extracted and saved. For the models from BioModels, we deduced possible time frames from figures in the publications or by visual inspection of the state trajectories and trying to determine reasonable settings. Afterwards, the adapted SBML models were grouped according to the first author and the year of the corresponding publication, the number of chemical species, and the number of reactions. If these four quantities coincided for multiple SBML models, we classified them as submodels of one benchmark model, in order to avoid biasing results by including the same model with minor variations multiple times. After this regrouping step, we were left with 259 benchmark models and 697 submodels.

The SBML models were then imported with AMICI (version 0.10.19, commit d78010f) leaving us with 148 benchmark models consisting of 455 submodels. The main reason for this loss is that AMICI did not support all features of SBML, such as rate rules or assignment rules for parameters or species. Those remaining models were also imported with COPASI, which reduced the overall number of accepted models to 147 benchmark models with 411 submodels. We assessed the correctness of the simulated state trajectories based on reference trajectories. For the models from JWS, reference trajectories were generated with the JWS built-in simulation routine, comprising 101 simulation time points along the trajectory. For the models from BioModels, we generated reference trajectories with the COPASI toolbox^19^ comprising 51 simulation time points, using the strictest error tolerances which still allowed a successful integration of the ODE system. As the COPASI simulator is probably the most widely used ODE solver implementation in the field of systems biology, we considered this the most reliable solution. Additionally, state trajectories were also compared by visual inspection.

All models were then simulated with AMICI using all 140 ODE solver settings from the main study and with COPASI using all 7 solver settings from the main study. We also tested whether a higher maximum number of ODE solver steps affected the result, but did not find a substantial impact. An SBML (sub-)model was assumed to be correctly simulated, if for each time point of each simulated state trajectory, the absolute or relative error (i.e., the mismatch between the reference trajectory and the simulation) were below a predefined acceptance threshold for at least one solver setting for either AMICI or COPASI. We investigated the number of accepted models in dependence of the acceptance threshold for various absolute and relative error tolerances (Supplementary Fig. S1). For too strict acceptance thresholds, all models were rejected, but when simulating with high accuracy, gradually increasing the threshold led to a plateau of accepted models for thresholds between $10^{-5}$ and $10^{-3}$. When increasing the acceptance threshold further, the number of accepted models grew until eventually all models were accepted. We interpreted the plateau of models at threshold values around $10^{-4}$ as those models which could be simulated correctly with sufficient accuracy and hence fixed the final acceptance threshold to a value of $10^{-4}$. Models were typically rejected, if the adaptation of the SBML file based on a corresponding SED-ML file did not work properly. This is the reason why the filtering step strongly reduced the number of SBML (sub-)models in the collection, while the number of benchmark models was less affected.

This procedure resulted in accepting a total of 259 SBML models, which were grouped to 142 benchmark models.

## Supplementary Information

Supplementary Information 1.
