# Supplementary material for: Benchmarking of numerical integration methods for ODE models of biological systems
Source: Sci Rep. 2021 Jan 29;11:2696. doi: 10.1038/s41598-021-82196-2 (PMC7846608; doi:10.1038/s41598-021-82196-2)
Supplement: Supplementary file 1 — Supplementary Information 1. [file 41598_2021_82196_MOESM1_ESM.pdf]

Supplementary Information to Benchmarking of numerical integration methods for ODE models of biological systems

Philipp Städter, Yannik Schälte, Leonard Schmiester, Jan Hasenauer, Paul Stapor

November 2020

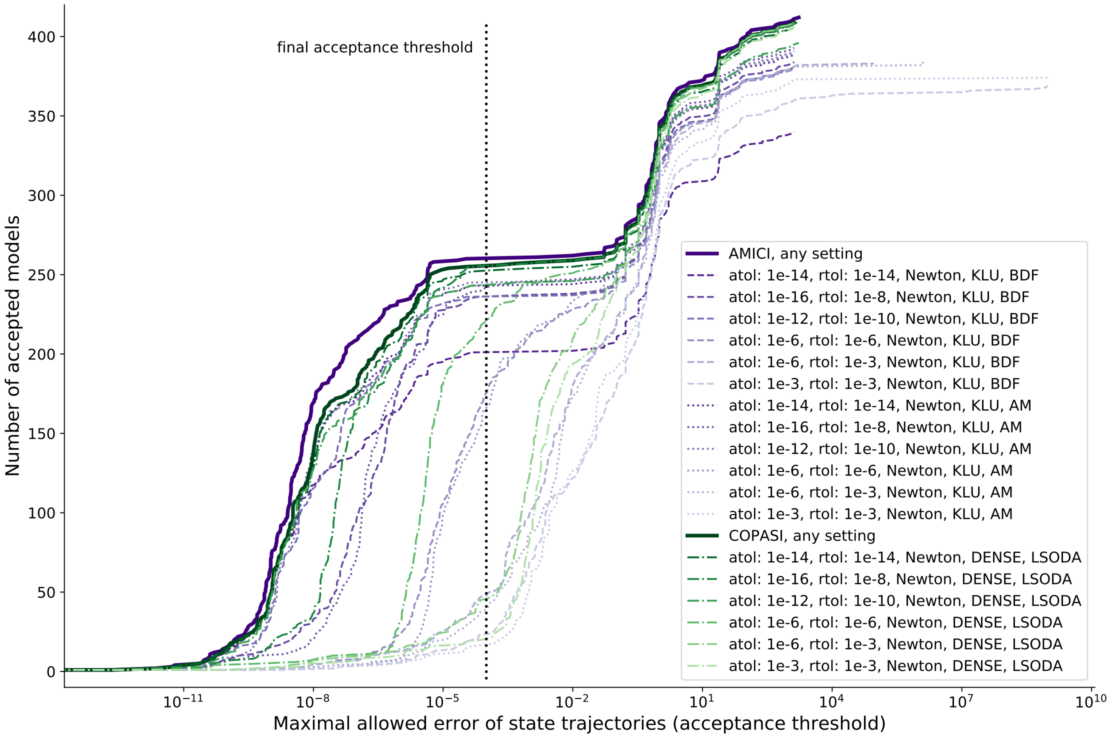

Figure S1: **Filtering of benchmark models by accuracy of model trajectories.** Number of accepted models for different acceptance thresholds, shown for a selection of solver settings (integration algorithms, linear and nonlinear solvers and combinations of absolute and relative tolerances). For a given solver setting, a model was regarded accepted if absolute or relative error of the simulated trajectories to reference trajectories were below a given acceptance threshold. Reference trajectories were either obtained from JWS or created with COPASI using the strictest tolerances which still allowed a successful model simulation. A model was finally regarded accepted, if it was accepted for any solver setting used in the study, for the shown final acceptance threshold.

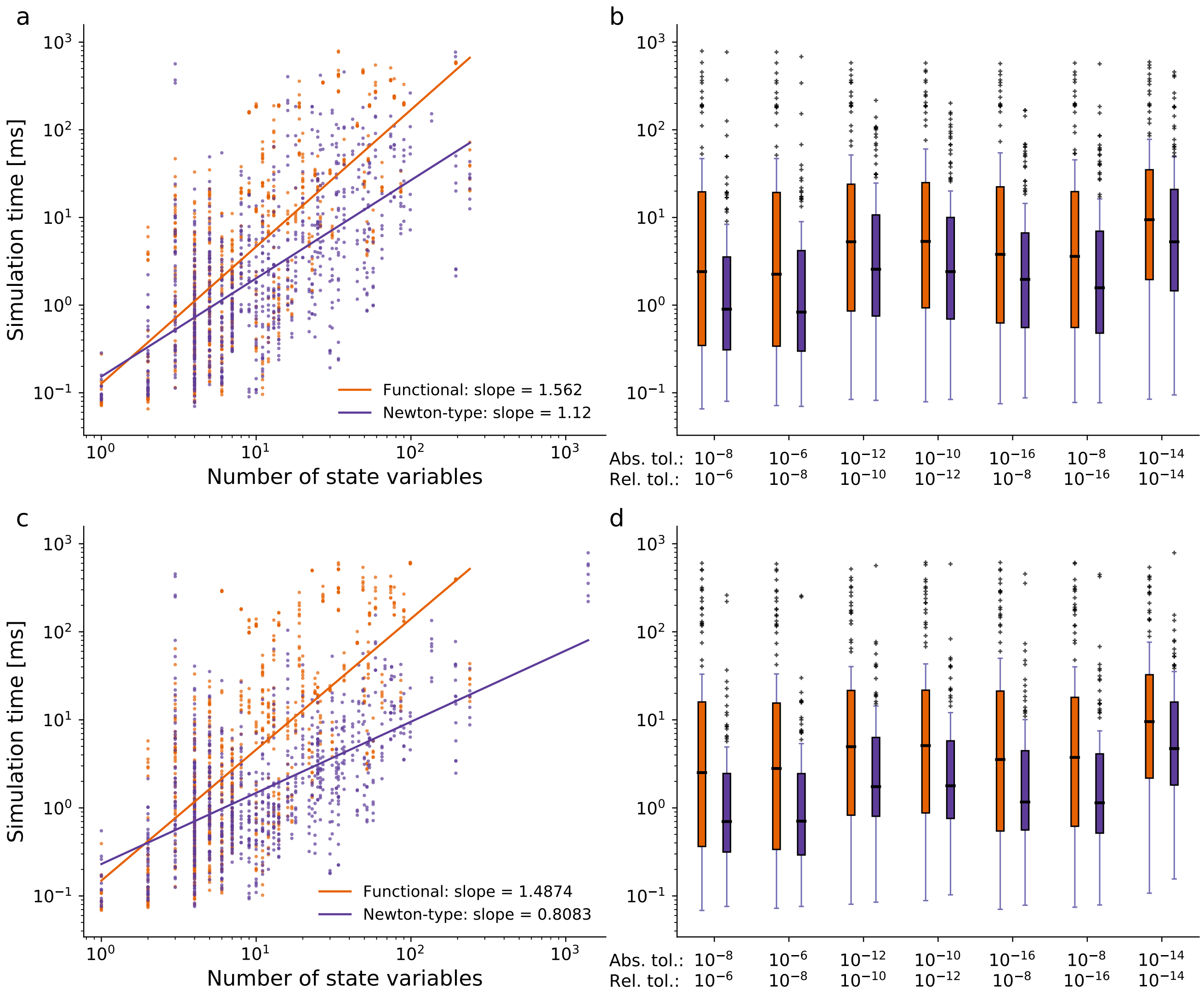

Figure S2: **Non-linear Solver.** Comparison between functional and Newton-type non-linear solver. In **a** and **b** shown for integration algorithm AM, in **c** and **d** for BDF. **a** and **c:** Scaling behavior of simulation times over numbers of state variables. Each point represents a model with a solver setting using the respective integration algorithm AM or BDF, the linear solver KLU, one out of the two non-linear solvers and one out of seven tolerance combinations. The accompanying linear regressions depict the overall scaling behavior of computation time with respect to the number of state variables. **b** and **d:** Computation time per tolerance combination. Visualization of the simulation time distribution using the respective non-linear solver in combination with one of the seven tolerance combinations, the respective integration algorithm AM or BDF, and the KLU linear solver.

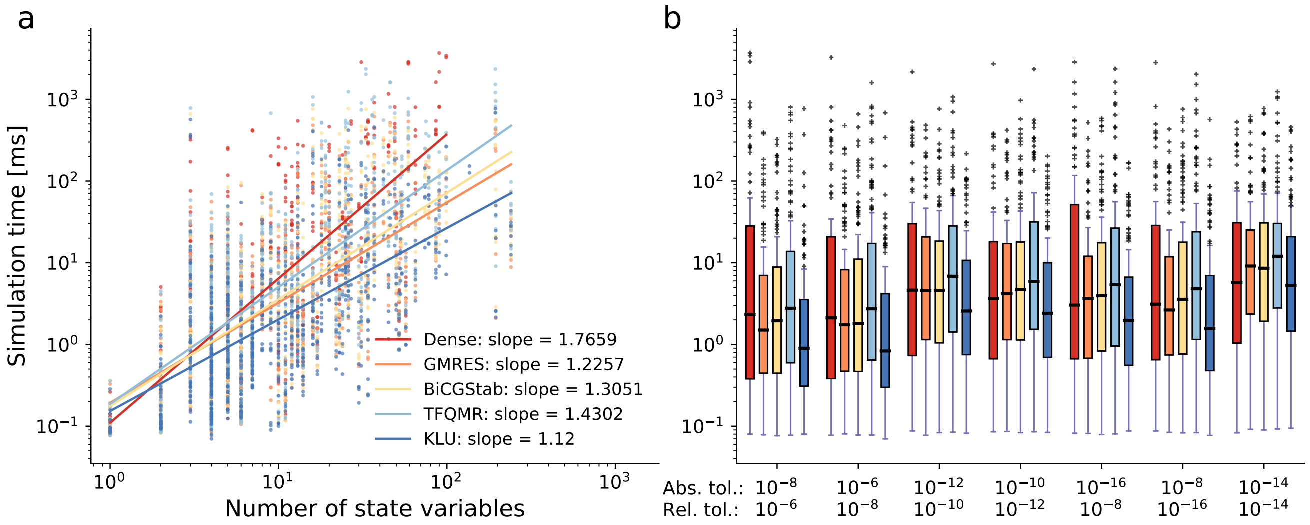

Figure S3: **Linear Solver using AM.** Comparison of the five CVODES linear solvers. **a:** Scaling behavior of simulation times. Each point represents a model with a solver setting using one out of five linear solvers, AM integration algorithm, Newton-type non-linear solver and one out of seven tolerance combinations. Colors are used to visualize the different linear solvers. The accompanying linear regressions depict the overall scaling behavior of computation time with respect to the number of state variables. **b:** Computation time per tolerance combination. Visualization of the simulation time distribution using a setting of one linear solver in combination with seven tolerance combinations, the AM integration algorithm and the Newton-type non-linear solver.

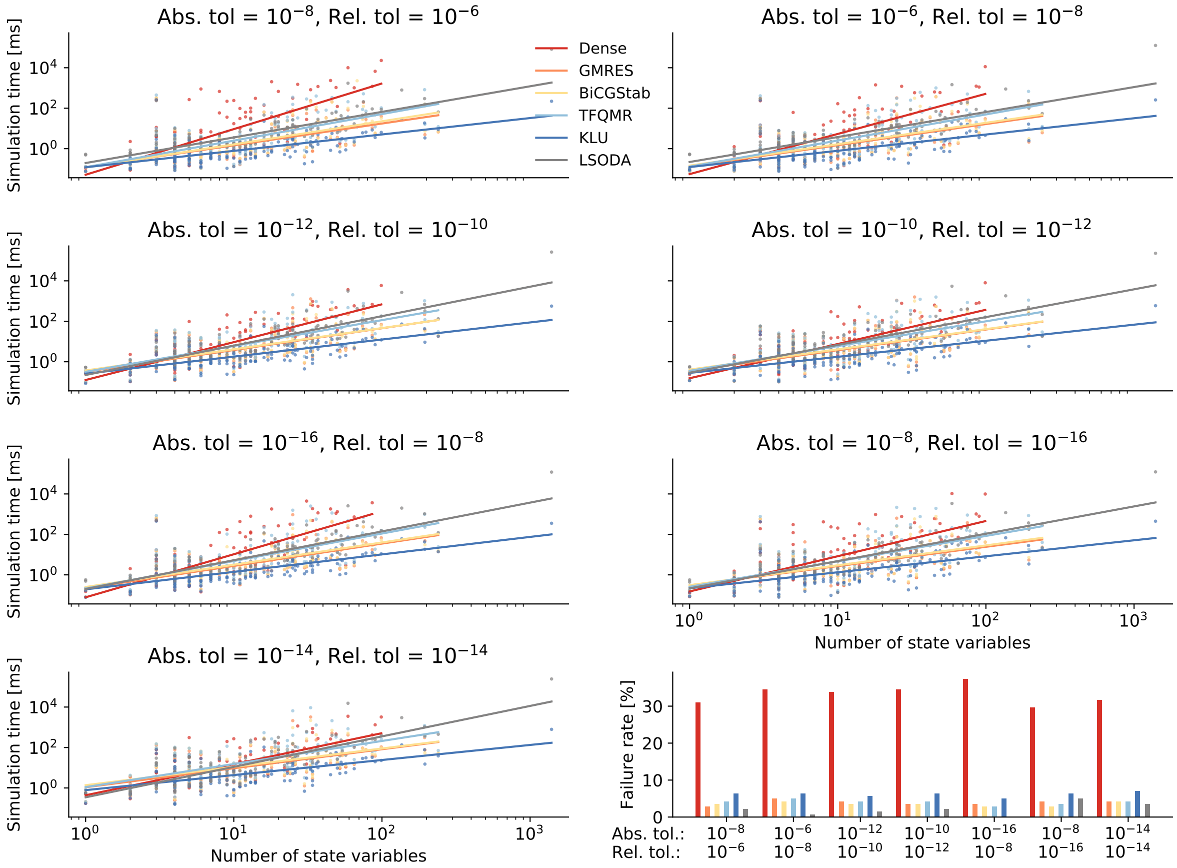

Figure S4: **Single linear solvers, BDF and LSODA.** Visualization of simulation times. Each color represents a linear solver setting with Newton-type non-linear solver and BDF integration algorithm, complemented by LSODA, and one of seven tolerance combinations. The accompanying linear regressions display the scaling behavior with respect to the number of state variables. The last plot shows the failure rates of the linear solvers for all seven tolerance combinations.

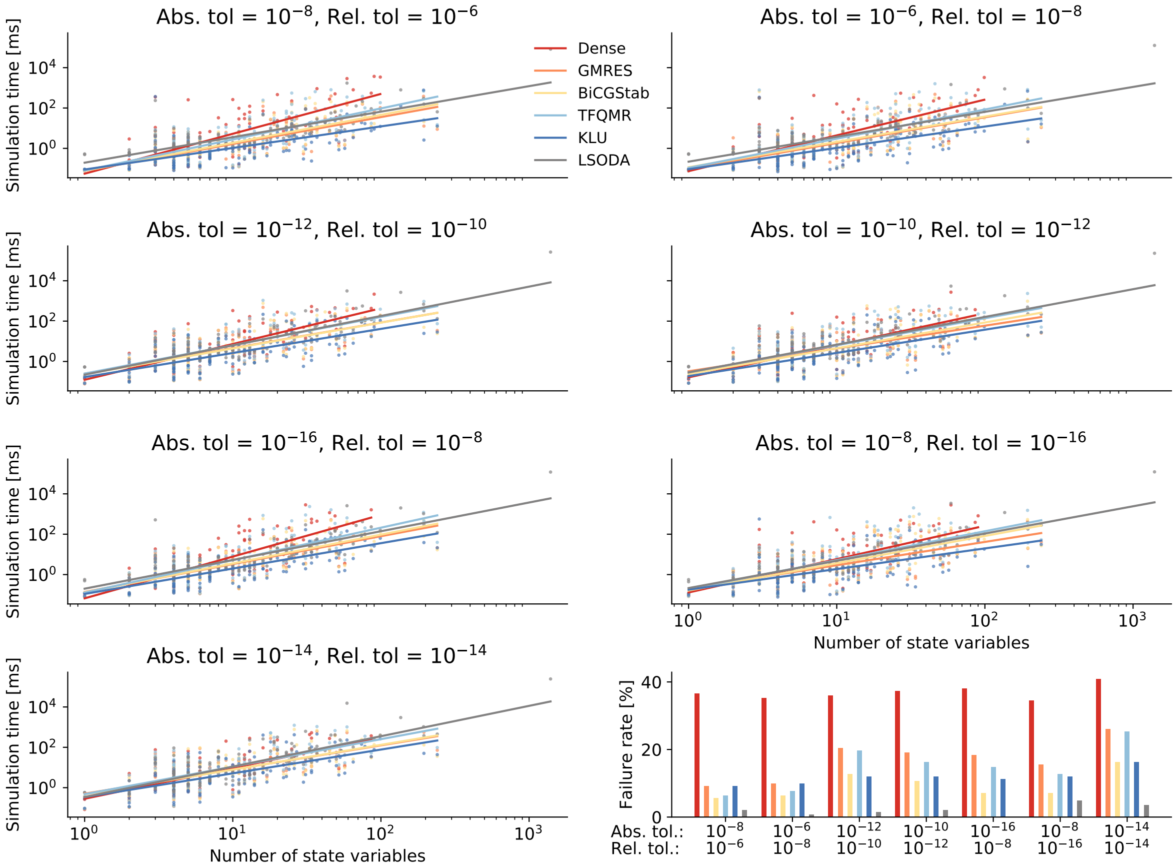

Figure S5: **Single linear solvers, AM and LSODA.** Visualization of simulation times. Each color represents a linear solver setting with Newton-type non-linear solver and BDF integration algorithm, complemented by LSODA, and one of seven tolerance combinations. The accompanying linear regressions display the scaling behavior with respect to the number of state variables. The last plot shows the failure rates of the linear solvers for all seven tolerance combinations.

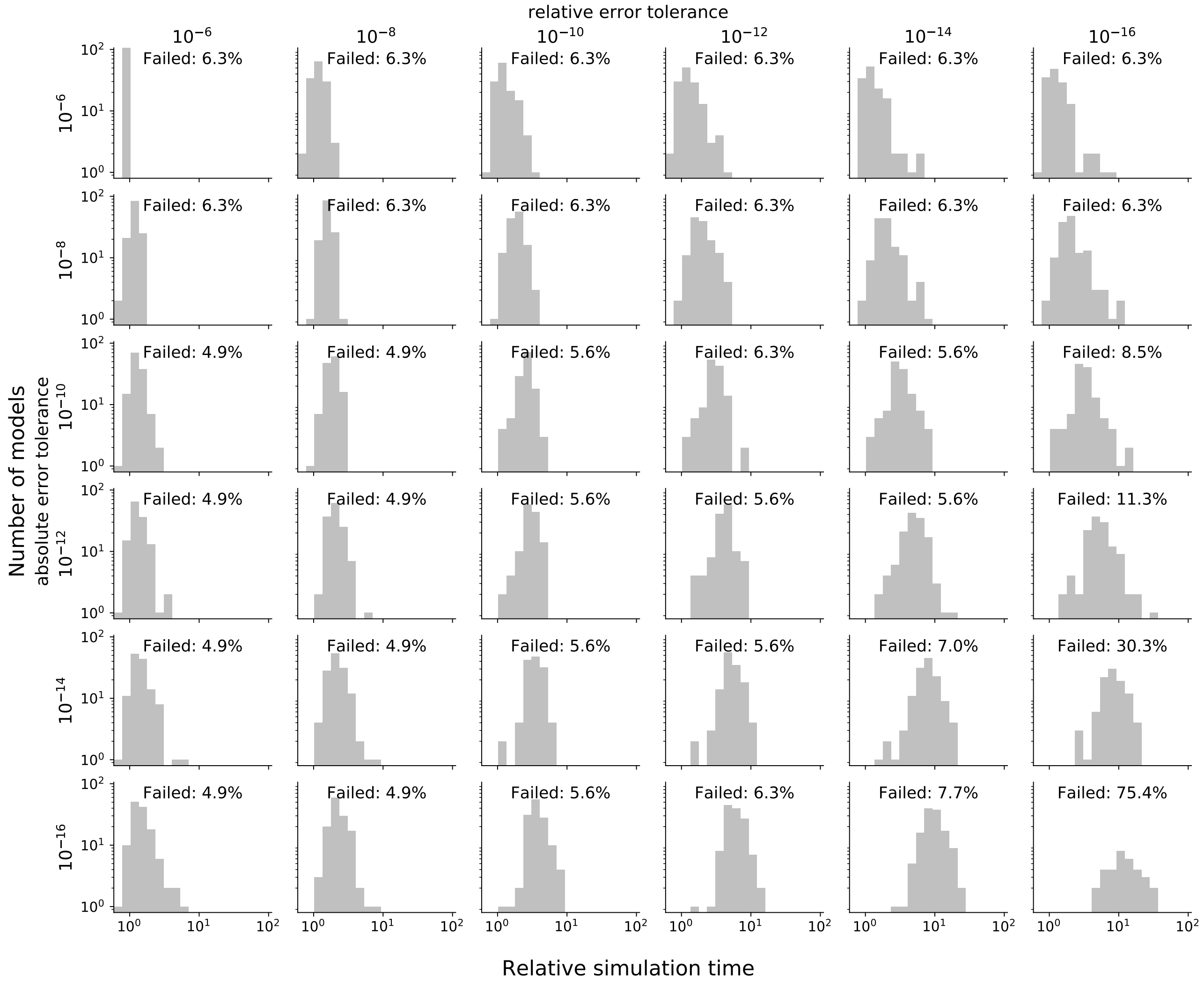

Figure S6: **Integration error tolerances, histograms.** Comparison of 36 error tolerance combinations composed of absolute and relative error tolerances. The simulation time of each model was normalised by the simulation time using the most relaxed combination (absolute error tolerance 10^{-6}, relative error tolerance 10^{-6}). The resulting simulation time ratios are depicted as histograms. The failure rate is displayed for each simulation setting at the top of the subfigure. All models were simulated by using the Newton-type non-linear solver, the linear solver KLU and the integration algorithm BDF.

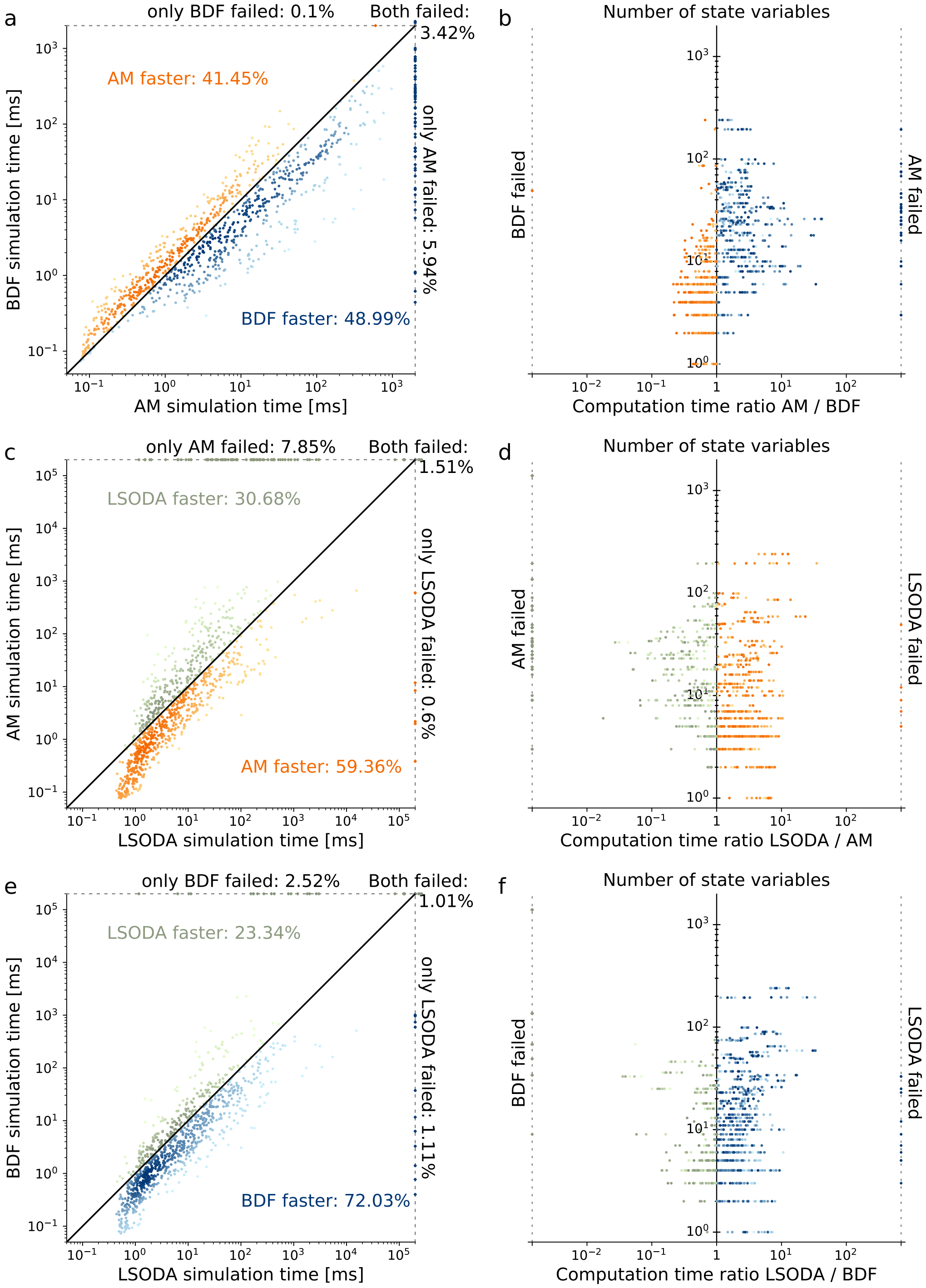

Figure S7: **Integration algorithms from CVODES using the linear solver BICGSTAB and LSODA. a:** Each scatter point depicts the computation time for a model using the AM (x-axis) or BDF (y-axis) algorithm with the Newton-type non-linear solver, the linear solver BICGSTAB and one out of the seven tolerance combinations, yielding seven points for each models. The scatter points on dotted lines on top and on the right denote failed simulations with only one or both integration algorithms. Darker colors represent a higher scatter point density. **b:** Computation time for AM divided by the computation time for BDF with respect to the number of state variables, using the color coding from subfigure a. **c:** Comparison of the LSODA algorithm (x-axis) with the AM algorithm (y-axis) with the same setup as in a. **d:** Computation time ratios for the LSODA devided by the AM algorithm, using the color coding from c. **e:** Comparison of the LSODA algorithm (x-axis) with the BDF algorithm (y-axis) with the same setup as in a. **f:** Computation time ratios for the LSODA devided by the BDF algorithm, using the color coding from e.

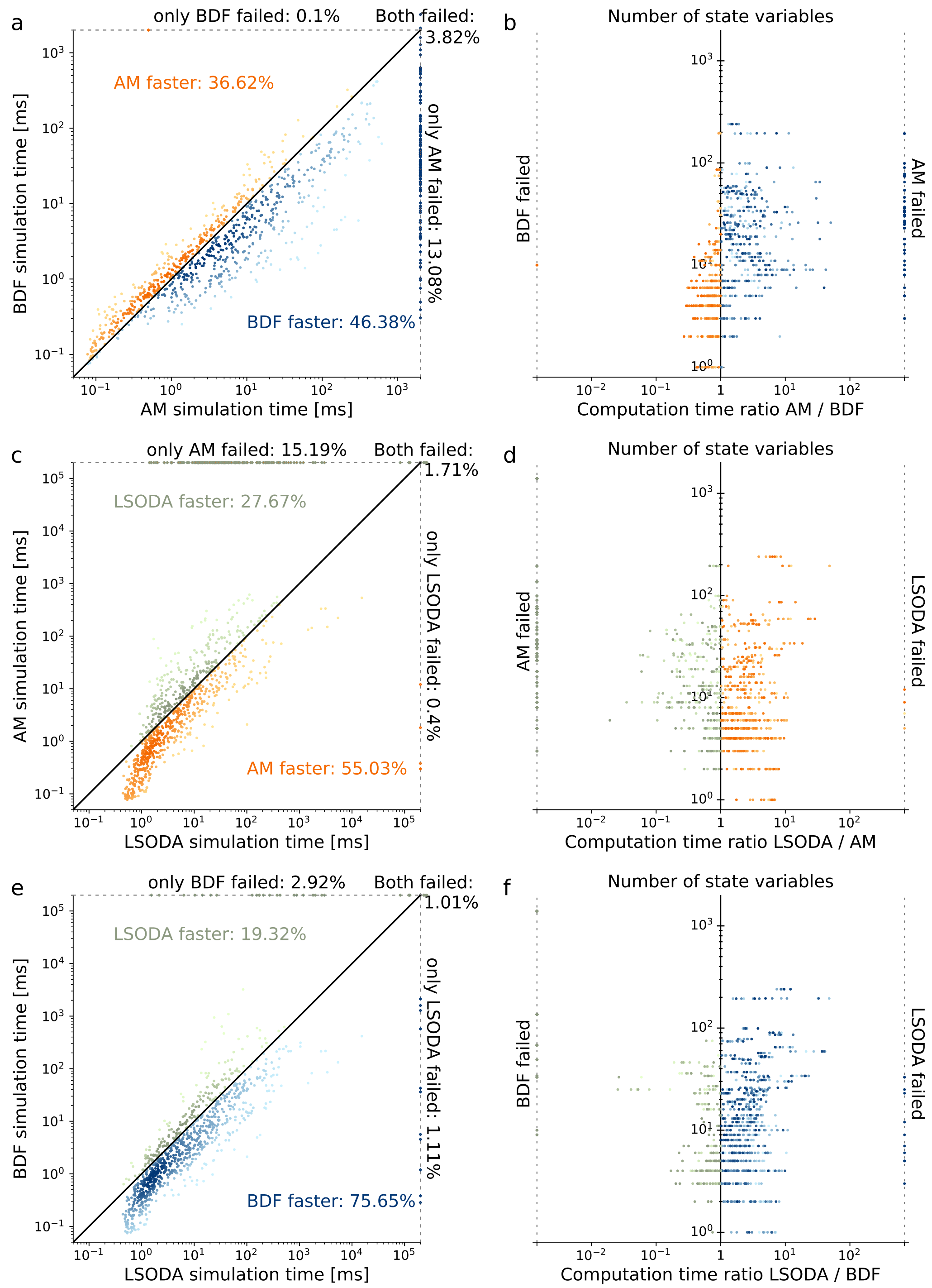

Figure S8: **Integration algorithms from CVODES using the linear solver GMRES and LSODA. a:** Each scatter point depicts the computation time for a model using the AM (x-axis) or BDF (y-axis) algorithm with the Newton-type non-linear solver, the linear solver GMRES and one out of the seven tolerance combinations, yielding seven points for each models. The scatter points on dotted lines on top and on the right denote failed simulations with only one or both integration algorithms. Darker colors represent a higher scatter point density. **b:** Computation time for AM divided by the computation time for BDF with respect to the number of state variables, using the color coding from subfigure a. **c:** Comparison of the LSODA algorithm (x-axis) with the AM algorithm (y-axis) with the same setup as in a. **d:** Computation time ratios for the LSODA devided by the AM algorithm, using the color coding from c. **e:** Comparison of the LSODA algorithm (x-axis) with the BDF algorithm (y-axis) with the same setup as in a. **f:** Computation time ratios for the LSODA devided by the BDF algorithm, using the color coding from e.
